# Recent Advances in the Excipients Used in Modified Release Vaginal Formulations

**DOI:** 10.3390/ma15010327

**Published:** 2022-01-03

**Authors:** Aikaterini Dedeloudi, Angeliki Siamidi, Panagoula Pavlou, Marilena Vlachou

**Affiliations:** 1 Department of Pharmacy, Division of Pharmaceutical Technology, School of Health Sciences, National and Kapodistrian University of Athens, 15784 Athens, Greece; dedeloud@pharm.uoa.gr (A.D.); asiamidi@pharm.uoa.gr (A.S.); 2 Laboratory of Chemistry-Biochemistry-Cosmetic Science, Department of Biomedical Sciences, University of West Attica, 28 Ag. Spyridonos Str., 12243 Egaleo, Greece; ppavlou@uniwa.gr

**Keywords:** novel vaginal excipients, co-polymers, mucoadhesive, bio-adhesive, thermosensitive, vaginal drug delivery, novel vaginal formulations, vaginal films, vaginal nanomedicines

## Abstract

The formulation of an ideal vaginal drug delivery system (DDS), with the requisite properties, with respect to safety, efficacy, patient compliance, aesthetics, harmonization with the regulatory requirements, and cost, requires a meticulous selection of the active ingredients and the excipients used. Novel excipients defined by diversity and multifunctionality are used in order to ameliorate drug delivery attributes. Synthetic and natural polymers are broadly used in pharmaceutical vaginal formulations (solid, semi-solid dosage forms, implantable devices, and nanomedicines) with a promising perspective in improving stability and compatibility issues when administered topically or systemically. Moreover, the use of biopolymers is aiming towards formulating novel bioactive, biocompatible, and biodegradable DDSs with a controllable drug release rate. Overviewing vaginal microenvironment, which is described by variable and perplexed features, a perceptive choice of excipients is essential. This review summarizes the recent advances on the excipients used in modified vaginal drug delivery formulations, in an attempt to aid the formulation scientist in selecting the optimal excipients for the preparation of vaginal products.

## 1. Introduction

Drug dosage forms involve excipients to offer properties to the active substance, such as physical stability, enhanced solubility, protection from microbial contamination, delivery optimization, and improved pharmacokinetics and/or bioavailability. The appropriate dosage form design involves consideration of the physicochemical and biological aspects of the active substance and the excipients, as they all must be compatible with each other [1]. The most common routes of drug administration are the oral route for achieving systemic effects and the topical for achieving local effects. Safety and efficacy of vaginal administration have been well documented for local, and systemic effects. Due to its large surface area and high blood supply, the requisite active substances can be easily absorbed through the epithelium of the vagina into the blood stream and exert their effects [2].

This review examines the utility of novel excipients in formulating vaginal delivery systems. The physicochemical properties of various synthetic and natural co-polymers were studied, and the release rate for drug-loaded formulations and the disintegration time for drug-unloaded formulations were also evaluated. In more detail, this review probes crucial attributes in selecting innovative excipients intended for modified release vaginal formulations which have been investigated and utilized in the last 15 years. The selection of research studies was conducted under the aspect of fabricating multifunctional pharmaceutical formulations by incorporating novel excipient combinations, targeting in patient compliance and side effects improvements characterized by safety and efficacy parameters.

## 2. Anatomy and Physiology of the Vagina

The vagina is located in women between the rectum, the urethra, and the bladder. It is a thin-walled fibromuscular tube that is extending from outside the body to the cervix [2,3,4]. The dimensions of the vagina are crucial factors that affect drug administration and are characterized by an interindividual variability, which is modified in an age-related manner. Thus, its average length in women of reproductive age is 6–8 cm for the front wall and up to 14 cm for the back wall, including the length of the cervix [5]. Moreover, the average vaginal surface area is calculated to be ca. 87.46 cm^2^ (range: 65–107 cm^2^) [6] and is significant for the absorption of administered active substances [7].

There are four distinct anatomical regions, which are observed in the lower part of the female genital tract: the introitus (covered by a keratinized epithelium), the vaginal epithelium (covered by a nonkeratinized stratified squamous epithelium), the ectocervix, (covered by a mucosal layer), and the endocervix (columnar epithelium with numerous glands) [2,3,4]. The physiological cervical stratified squamous epithelium is formed by distinct layers of epithelial cells, which can be classified into different classes based on the stage of maturation: basal, parabasal, intermediate, and superficial. Throughout maturation, various cells from the basal layer move gradually towards the superficial layer, obtaining a flatter shape. However, superficial layers of the cervix, which are closer to the vaginal lumen, are mainly characterized by tight junctions [8].

Furthermore, the thickness of cervical squamous epithelium is variable (0.2–0.5 mm) and is directly related to age. After menopause, cytoplasmic volume condensation and epithelium atrophy is observed [9]. It is essential that the structure of the epithelium is considered as the crucial site for the most effective and targeted drug delivery of the active substances, the permeability and solubility properties of which determine the final drug delivery efficacy (topical or systemic action) across these tissues and the toxicity profile [8].

## 3. Vaginal Drug Delivery

The anatomy and physiology of the vagina can affect and provoke challenges in achieving local or systemic drug delivery [8,10]. The most common drugs that are usually administered in the vagina are related to the treatment of vaginal infections (antimicrobials/antifungal), spermicides or contraceptive agents, and active ingredients related to hormone replacement therapy, induction of labour, and interruption of pregnancy [11,12]. There is a plethora of formulations used for vaginal administration including solid dosage forms (suppositories and tablets), semisolids (creams and gels) and other forms such as intravaginal rings, films and foams [13]. Plenty of variables determine the selection of vaginal administration, which may depend on drug properties to clinical requirements for acceptability by the patient [14].

Several advantages are provided by vaginal DDSs [2,12,14,15]. The vaginal route is easy and convenient due to the simple administration of the formulations. It is an accessible and noninvasive route, since it does not cause tissue damage and potential infections related with parenteral administration. Moreover, drugs which have a systemic action and are administered vaginally are easily absorbed due to vaginal high vascularity, which allows adequate blood supply and, consequently, they do not undergo from hepatic first pass metabolism [8,16]. Vaginal administration also minimizes many side effects which can be provoked by oral and parenteral drugs, such as those affecting the gastrointestinal tract and liver [16]. Other absorption pathways that can occur through this route are passive diffusion and active transportation, which are defined by permeability and absorption characteristics, such as the molecular weight (MW), the extent of ionization, the lipophilicity, and the dissolution properties of active substances [10].

Another significant advantage of the vaginal route drug administration is the specialized local effect of the drug when treating infections or preventing sexually transmitted diseases of the female reproductive system. Specifically, a high administered dose can be used in this area without causing significant adverse effects due to its limited systemic drug exposure compared to other routes (i.e., oral or parenteral) [10].

## 4. Factors That Affect Drug Absorption in Vaginal Epithelium

**Figure 1:**
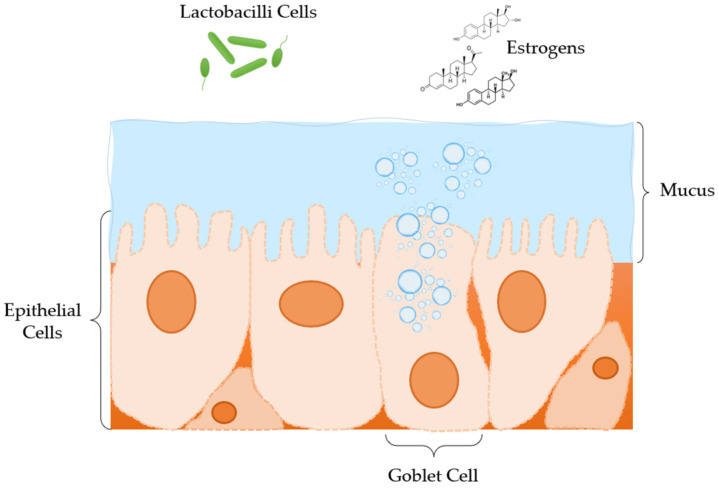
Anatomical and physiological features of the vagina microenvironment.

Physiologically, the vagina generates several obstacles in drug delivery due to its variability in potential structural form, which can affect the acceptability and the administration of certain vaginal formulations [10]. These variations are crucial to be considered from the pharmacological point of view, since drug administration and absorption are directly related with the physiology of the vagina. These factors are cervical mucus, vaginal epithelium thickness, fluid, microflora, enzymatic activity, and pH [7] (Figure 1).

During the menstrual cycle, the vaginal epithelium thickness is modified as a reaction to oestrogen levels changes, which are responsible for the increase of the epithelium thickness. Moreover, the vaginal epithelium is consisted of squamous cells, which have gaps in their connecting links that form a series of canals between adjacent cells, allowing the movement of molecules and electrolytes and the absorption of certain drugs [7]. Also, the thickness of the squamous epithelium varies (0.2–0.5 mm) and is substantially affected by age [17]. Vaginal mucosa layer is related to oestrogen levels that are spatio temporally regulated, according to hormone cycle fluctuations that are depended by age. Changes in vaginal mucosa levels occur during puberty, along with adrenal and gonadal maturation, and reach a maximum in thickness and glycogen volume during ovulation. Mucus thickness and vaginal fluid secretion modifications are adapted during pregnancy, due to hormonal alterations. In menopause and postmenopause time periods, cervico-vaginal secretions diminish and become sparse [18,19]. Thus, drug permeability, through the vaginal surface, can affect drug distribution and pharmacokinetics and, as a result, the drug’s efficacy and resulting toxicity levels [8,12,14].

The vaginal epithelium is coated in a mucous layer, which consists of water, a matrix of mucins (glycoproteins with high MW), enzymes, plasma proteins, amino acids, lipids, cholesterol, and a number of inorganic ions [20,21,22]. Mucous secretions (composition, quantity, and physical characteristics) depend on the menstrual cycle, as oestrogens affect their production. They are produced from goblet cells in the epithelium of the endocervix and Bartholin glands. However, the squamous epithelium of the vagina and ectocervix are not involved in the production of mucus. Furthermore, this mucus layer is structurally analogous to a complex net of tangled microfibers with multiple coarsened folds, which enhance the absorption surface. Specifically, during ovulation, the amount of mucus secretion is augmented, creating larger, thicker, and more viscous pores, leading to an increase in the total volume of vaginal fluid, a higher pH, and mucin content. Hence, mechanical, and structural features of vaginal mucus have a significant impact on drug targeting and release rate, affecting the pharmacodynamics of certain drugs [10].

Another significant factor to drug delivery is the vaginal fluid, which is produced by the mucous membranes of the endometrium and then accumulates inside the vagina covering the vaginal epithelium [7]. The major function of this fluid is to coat the vaginal mucosa and to shelter the deeper tissues against a possible entry of pathogens [8]. The vaginal fluid is complex; it is transudated from blood vessels that surround the vagina, goes through the vaginal wall, it mixes with secretions from the sebaceous, sweat, and Skene glands, and also contributes to the endometrial and oviductal fluids in the vagina [8,23]. Although its main components are cell mucus and elements associated with the innate microflora of the vaginal epithelium, it also contains various components, such as amino acids, proteins, carbohydrates, enzymes, enzyme inhibitors, ions, lipids, and possibly macrophages, lymphocytes, plasma cells, Langerhans cells, eosinophils, and mastocytes [7]. The fluid layer on the epithelium and the enzyme activity in the vaginal fluid has been identified as a significant barrier to drug delivery and absorption and plays a significant role in ensuring that the active substance reaches its target [8]. Moreover, the release rate profile of drugs administered by this route and the residence time and bioadhesion of the formulation can be modified by dynamic changes in the volume and composition of the vaginal fluid [8,10].

One more aspect that influences drug delivery in the vaginal fluid is its pH. The normal pH value in an adult woman is ~3.5–5.0, due to commensal *Lactobacillus* sp., which produces lactic acid from the glycogen from the sloughed epithelial cells of the mucosa. The deposition of glycogen on the vaginal epithelium depends on the presence of oestrogens. Nevertheless, glycogen reduction is observed in postmenopausal women. In more detail, after birth, microbes or normal flora colonize vagina, which are responsible for lactic-acid production. In early childhood neutral or alkaline pH is prevalent and lactic acid microbial population decreases. During puberty, periodical alterations in glycogen and increase in lactic-acid microbiome are observed. Throughout reproductive years, menstrual cycle defines changes in cervico-vaginal secretions and pH variation. However, during postmenopause, cervico-vaginal secretions decrease, and vaginal pH becomes more alkaline [19].

Low pH value (3.5–5.5) of the vagina provides an innate protection from pathogens [8,18,24], whilst higher values are indicative of infection. Drug ionisation potential depends on micro environmental pH of the anatomical area. Vaginal administered drugs are classified in weak bases (>50%, pKa = 8.5–10.5) and in weak acids (~40%, pKa < 5.5) and the absorption rate varies and depends on vaginal pH provoked by occurring illness [25]. The specific vaginal pH range has to be carefully examined during the formulation procedure in order to ensure the stability and the release rate of the active substances [7].

Moreover, normal vaginal flora has a crucial role in defining the environment, in which active pharmaceutical ingredients (APIs) would be released. Vaginal microflora consists of cocci and bacilli species of Gram-positive and Gram-negative bacteria and small amounts of anaerobic microorganisms [23,26]. The presence of different species of microorganisms depends on the physiological conditions of the vagina, which are significantly related with age, day of menstrual cycle, pregnancy, menopause, infections, and douching practices. Thus, the presence and the contribution of physiological vaginal microbiota in maintaining a healthy vaginal environment, but also in creating barriers to drug delivery have to be taken into account, when designing vaginal DDSs [8,27,28].

Several enzymes are present in both vaginal and cervical secretions, and their activity may vary during the menstrual cycle. However, their activity has not been fully determined, as yet [7]. Vaginal enzymatic potency is defined as moderate. Thus, drug degradation has to be examined profoundly when active substances are peptide-based. Aminopeptidases, dipeptidyl peptidases, and dipeptidyl carboxypeptidases are the most abundant enzymes in the outer layers of vaginal mucus and are involved in enzymatic degradation processes of peptide and protein drugs; consequently, affecting drug absorption and therapeutic levels. Although, the identification and quantification of vaginal enzymes are not characterized totally, lysozyme is also present in human cervical mucus [29]. Vaginal enzymes may also affect the drugs’ stability and their passage through the vaginal epithelium [14]. A study of Olmsted et al., has shown that viscosity of vaginal fluid samples from women with bacterial vaginosis was lower than the physiological. Alteration in viscosity was provoked due to decreased enzymatic stimulation in the pathophysiological vaginal environment [30].

## 5. Excipients Used in Modified Drug Release Pharmaceutical Dosage Forms for Vaginal Administration

**Figure 2:**
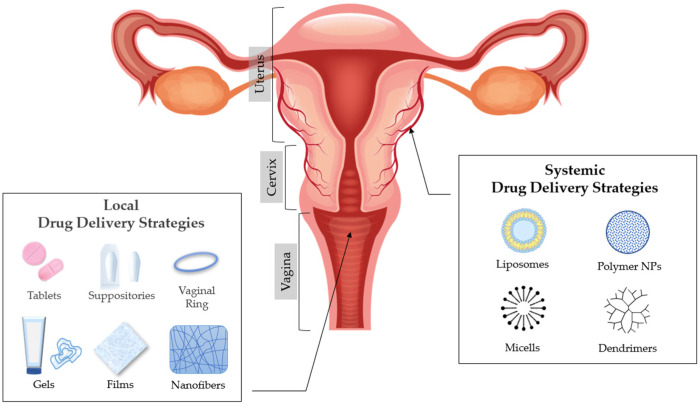
Anatomy of the vagina and drug delivery strategies.

A proper pharmaceutical formulation has to be described by quality characteristics, such as safety, efficacy, stability, and acceptability. However, many considerations have to be taken into account when developing a drug product for vaginal administration [8]. The choice of the dosage form depends on the physical and chemical characteristics of the drug, its mechanism of action, the target of the formulation and last but not least patient comfort [8,31]. Many pharmaceutical formulations have been used for drug delivery to the vagina. Despite the fact that traditional formulations, such as vaginal creams [32,33], gels [34,35,36], tablets/capsules [37,38] and suppositories [39] have a prevalence, there are other more novel dosage forms such as films [40] intravaginal rings [41,42,43] and nanomedicines [14] (Figure 2).

### 5.1. Excipients Used in Matrix Tablets for Vaginal Administration

The most common type of vaginal DDSs are matrix tablets [38], in which the active substance is uniformly distributed in a matrix of hydrophilic polymers. When hydrophilic polymers interact with the vaginal fluid, they create an *in situ* gelling effect, through which the drug diffuses. The release rate of the active substance from matrix type tablets depends on the type, ratio, particle size, and solubility of the polymer, the solubility and particle size of the drug, and the geometry of the matrix. The mechanisms of erosion and diffusion take place simultaneously in the dissolution of matrix type tablets and determine the release of the active ingredient. The dominant release rate mechanism is determined by the solubility properties of the drug [7]. Polymeric matrix type tablets offer several advantages. First, their manufacturing process is facile and inexpensive and a variety of polymers can be used in order to define a desirable release rate (immediate, sustained release rate, etc.). Furthermore, as they are solid formulations, they can be simply applied by the patient and show a major acceptability and convenience in use. Their significant stability ensures accuracy in the administered dose and a great capacity to control drug release according to therapeutic needs [38]. Vaginal tablet formulations require excipients, such as diluents, binders, disintegrants, glidants, lubricants and antiadherants.

Amongst the excipients used for modifying drug release, chitosan (CS), a cationic natural polymer, is widely used in pharmaceutical applications, as it possesses attractive biological properties, such as biocompatibility, biodegradability, non-toxicity, and physiological inertness [44]. CS is a polymer formed by the partial deacetylation of chitin. Systems containing this excipient have shown sustained drug release, mucoadhesive properties, intrinsic antimicrobial activity and immunostimulant capacity. It has been used in the development of vaginal formulations, such as tablets, films and gels, and nanomedicines. More particularly, scientists have investigated chitosan alone but also in combination with pectin and locust bean gum as excipients used in the formulation of mucoadhesive vaginal matrices for the sustained release of tenofovir. The results revealed that the association of chitosan and pectin can generate polyelectrolyte complexes and produced a robust system with mucoadhesion residence time on vaginal mucosa and a controlled release of tenofovir for four days, the time corresponding to vaginal turnover [45]. Two-layered tablets containing microencapsulated *Lactobacillus* spp. bacteria have been developed for the treatment against vulvovaginal infections. The excipients used for the fast release layer included, amongst others, lactose and starch, while polyacrylic acid derivative (carbopol^®^ 934) and chitosan were used in the slow-release layer. The results indicated that chitosan prolonged the release up to 24h [46]. Researchers have also evaluated chitosan and synthesized S-protected chitosan (with the aid of *N*-acetylcysteine and 6-mercaptonicotinamide) metronidazole tablets with the latter system showing prolonged residence time on vaginal mucosa and controlled release of the embedded drug. Moreover, drug loaded tablets presented slower dissolution rate due to lipophilic properties of metronidazole. S-chitosan is defined as a multifunctional excipient not only for significant mucoadhesive properties but also for oxidative stability enhancement of tablets and antimicrobial properties [47]. 

Hydroxypropylmethylcellulose (HPMC) and sodium carboxymethylcellulose (NaCMC) are water soluble derivatives of cellulose with valuable applications in pharmaceutics. Researches have prepared vaginal tablet formulations of clotrimazole, a widely used antifungal agent for vaginal mycotic infections, by direct compression, using bioadhesive polymer mixtures of HPMC/NaCMC that showed mucoadhesive strength and modified drug release. HPMC, NaCMC, and GG are hydrophilic polymers, characterized by considerable mucoadhesive properties. Subsequently, contributing to swelling effect of the matrix and drug diffusion processes. It was shown that hydrogen bond interactions between NaCMC and HPMC resulted in CMC swell. Moreover, HPMC, as a non-ionic with longer polymeric chains polymer, when used in combination with NaCMC, contributed to higher mucoadhesive effects and more delayed release of clotrimazole. In detail, formulations with NaCMC/HPMC mixture at concentrations of 40 and 20%, respectively, exhibited satisfactory swelling and bioadhesiveness, thus promoting sustained effect in the vagina (72% drug released over 12 h) In contrast, HPMC/GG formulations were characterized by higher viscous gel layer around the tablet providing greater swelling effect of the formulation [48].

### 5.2. Excipients Used in Solid Dosage Forms for Vaginal Administration: Ovules and Vaginal Suppositories

Scientists have also explored alternatives to vaginal tablets. Ovules and vaginal suppositories have been formulated for topical delivery. The formulation of such products requires a base (oily/emulsifying/aqueous), where the API will be placed in, and in some cases antioxidants and/or preservatives are also used [15]. The composition of the suppository base has an important role in drug release rate and extent. Suppository bases can be classified according to their composition and physical properties to oleaginous (fatty or hydrophobic) and water-soluble bases. Oleaginous bases include theobroma oil (cocoa butter) and synthetic triglyceride mixtures (hydrogenated vegetable oils such as palm kernel oil and cottonseed oil). On the other hand, water soluble bases are those containing glycerinated gelatin or polyethylene glycol (PEG) polymers, releasing the drug when dissolved in the aqueous body fluids. Glycerinated gelatin suppositories tend to disperse slowly in mucous secretions providing prolonged drug delivery, while PEGs that are available in a large range of MW can be formulated in a wide range of hardness and therefore, dissolution time [49].

HPMC is a non-ionic, semi-synthetic cellulose derivative, used in pharmaceutics as a gelling agent and for drug release control [50]. HPMC has been utilized in combination with vaginal suppository bases in order to alter drug release rate. In a recent study, fluconazole vaginal suppositories were prepared by using three different combinations of polymers (agar and HPMC) and/or suppository bases (glycerol-gelatin base, cocoa butter, and bees wax). Results indicated sustained drug delivery, especially for the suppositories prepared by using agar and HPMC mixtures, as they showed 80% drug release over 12 h [51].

In another study, researchers have formulated new polyethylenimine (PEI) (of low MW and linearity) based suppositories for intravaginal gene delivery for the prevention and treatment of cervical cancer. The success of gene therapy depends on an effective delivery system, which is biocompatible to the delicate vaginal epithelial mucosa cells. The formulation consisted of two important parts; the DNA-PEI complexes, which were associated with delivery of DNA-plasmids for the gene targeted-therapy, and the hard fatty base suppocire^®^, which contributed to the formulation’s durability and the sustained release of DNA-PEI composites [52]. PEI, is a cationic polymer, with repeated units consisted of the amine group and two carbon aliphatic CH_2_CH_2_ spacers. MW and structure of PEI have been proven critical in determining its toxicity; with high MW and branched PEI to be more toxic than the low MW and linear structure, and PEI-DNA complexes less toxic than free PEI, due to polymer aggression. Lower MW linear PEI has been proven to be biocompatible in vaginal mucosa and effective non-viral vector for gene delivery and cell-targeting [53], representing a key element for the sustained delivery of DNA-plasmids [52].

### 5.3. Excipients Used in Semi-Solid Dosage Forms for Vaginal Administration: Gels

Gels are semisolid systems that consist of two components (a liquid and a solid) [8]. When these components are homogenized, the solid component traps the liquid, creating a gelling effect and consequently producing a solid-liquid mixture. According to the polarity of the liquid component, gel systems are categorized in hydrogels, which contain a polar solvent (such as water) and organogels, where the liquid component is apolar (such as vegetable oils). Hydrogels are used in pharmaceutical industry for the administration of active substances on skin and/or mucous membranes. They are widely accepted by patients for being refreshing, hydrating, and easy in application and in removal. Therefore, their hydrophilicity creates a difficulty in drug release and consequently in absorption through the lipophilic skin barrier. As far as organogels are concerned, they outline a great ability to cross the skin barrier. However, they are not highly acceptable from patients, due to their greasy texture [54,55,56]. Furthermore, combinations of hydrogels and organogels, in defined ratios, conduct to the formation of a bigel, in which lipophilic and hydrophilic drugs can be included in the same dosage form for topical or transdermal administration [7]. Gels offer various advantages when used as vaginal DDSs. Firstly, these pharmaceutical forms are easy and convenient in application, so treatment adherence is significantly improved with a great acceptability from patients [57,58,59,60]. Moreover, their ability to create a thin layer over the vaginal mucosa characterizes them with an optimal and rapid profile of release and absorption. Another significant advantage of gels is that their use is not only effective for treatment but also for prevention of infections, due to their ability to cover a greatly large surface of the vaginal mucosa; thus, offering a remarkable physical barrier against external pathogens. Furthermore, the cost of their production is low, compared to more complex dosage forms, such as vaginal rings [10,61,62]. However, gels present several disadvantages. Many trials have shown that vaginal gel utilization can cause leakage, wetting and/or messiness [63,64,65,66,67]. Another fact to be considered in semisolid formulations, is the difficulty in ensuring the uniformity of dose. Therefore, this creates a limitation in active substance selection, as it must have a sufficiently broad therapeutic margin in order to assure its pharmacological effectiveness. Another difficulty that occurs, is that gels have little efficiency in controlled drug release, consequently, may not be appropriate DDSs for repeated administration treatments [61]. Vaginal gel formulations require excipients such as a vehicle, gelling agents, humectants, and preservatives.

It has been recently proposed that the employment of multifunctional polymers, such as poly(acrylates), chitosans and their thiolated derivatives, may improve mucoadhesion, penetration enhancement, and enzyme-inhibiting properties [68]. Researchers prepared chitosan-based gels and evaluated the employment of chitosan citrate as multifunctional polymer having mucoadhesive, penetration enhancing and protease inhibition properties. Citrate, due to its chelating properties, is able to bind divalent cations such as calcium, which is involved in the regulation of gap and tight junctions. The results obtained suggested that the citrate salt of chitosan can be suitably employed in gels for the modified vaginal administration of poorly permeable drugs, such as acyclovir and ciprofloxacin HCl [69].

From a different point of view, researchers have developed vaginal bioadhesive gels for the controlled release of tenofovir containing guar gum hydrogel and sesame oil, adding Span^®^60 and Tween^®^60, as surfactants. A bigel microstructure was produced, which was able to control drug release for several days, remaining attached to the vaginal mucosa for at least the same time period. The presence of Span^®^60 in the systems allowed the sesame oil to be dispersed in droplets inside the guar gum hydrogel and to produce an emulgel. The addition of the second surfactant (Tween^®^60), compatible with the first, gave rise to bigels, consisting of a guar gum hydrogel and a Span^®^60-sesame oil organogel. In this system, which contained a defined proportion of each gel, a crosslinking occured between the three-dimensional structure of the hydrogel and the organogel. This resulted in a lower degree of swelling, more sustained release of the drug and longer bioadhesion time, due to the slower disintegration of the system in the presence of vaginal fluid [70].

Furthermore, a group of scientists developed a metronidazole microsphere-loaded bioadhesive vaginal gel with long residence time at target site and a sustained release profile. The microspheres were prepared by the solvent evaporation method using poly-*ε*-caprolactone (PCL), while the bioadhesive gel was prepared using Carbopol 934P and HPMC K4M. Results indicated sustained release of >10 h, showing suitability for the sustained delivery of the drug. The observed bioadhesive strength suggested enhanced retention of the applied gel in the vaginal sites, which is helpful in sustained release as well as for effective drug absorption [71].

### 5.4. Excipients Used in Films for Vaginal Administration

Solid and semisolid conventional vaginal dosage forms usually show a short residence time on the vaginal mucosa especially in disease condition due to added vaginal secretions. Therefore, the therapeutic effect is available only for a short period of time requiring frequent dosing and resulting in poor patient compliance. A method to overcome this, is the in-situ gel forming films. Polymeric films are safe and effective and can be used for vaginal administration of various drug candidates. Vaginal film formulations require excipients such as a film former plasticizer (polymer), humectant(s), and a solvent [49].

As an alternative formulation to solids and semi-solids, researchers used hot melt extrusion to manufacture a dapivirine vaginal film [72]. In another study, polymeric films were developed for the vaginal delivery of the highly potent and non-toxic, dual-acting HIV non-nucleoside reverse transcriptase inhibitor (NNRTI) pyrimidinedione, IQP-0528. Results revealed that the increase of PVA concentration is proportionally correlated with retention time at the target area and identified the formulation as an effective method for drug delivery to the vaginal tissue. In detail, HPMC and PVA concentrations affected film rigidity and disintegration time. However, the use of PEG400 and glycerin did not have any impact on drug release rate [73]. Also, scientists have developed a novel bioadhesive vaginal film formulation of polystyrene sulfonate, a novel non-cytotoxic antimicrobial contraceptive agent for prevention of sexually transmitted disease using the solvent evaporation technique. The films formulated were colorless, transparent, thin, soft, and tough, dissolved rapidly in physiologic fluid to form a smooth, viscous and bioadhesive solution that could be retained in the vagina for prolonged intervals, with a release rate of 5 mg/mL for a 250 min time duration. Moreover, system capability in providing an initial fast film dissolution along with a simultaneous prolonged drug release rate, permits a patient-firendly applied therapy, devoid of compliance issues [74]. In another work, different vaginal films based on chitosan, hydroxypropyl methylcellulose, blends of these polymers and different contents of PEG as plasticizer containing tioconazole, were developed and thoroughly characterized to improve the conventional therapeutics of vaginal candidiasis. Mechanical properties, swelling, adhesiveness, morphology, antifungal activity, hemocompatibility, and cytotoxicity were evaluated. The system based on CS-HPMC with 40% PEG 400 as plasticizer showed fast and sustained antimicrobial activity and also the lowest swelling value. Additionally, this formulation produced no cytotoxic effects, showing that this film is a promising alternative dosage form for the treatment of vaginal candidiasis [75]. In another study examining the mucoadhesive potential of a controlled release tri-layered vaginal film formulation, consisting of chitosan and ethylcellulose, it was investigated that chitosan is capable of creating polyelectrolyte complexes (PECs) with other polymers. In detail, when amine moieties of chitosan and anionic polymers, such as xanthan gum, karaya gum and pectin, coexist in vaginal acidic pH conditions, electrostatic interactions are taking place and PEC complexes are formed *in situ*, permitting tenofovir’s release through swelling mechanisms [76]. Moreover, when chitosan is formulated in combination with other materials (e.g., PEG and HPMC) can provoke less cytotoxic effects ameliorating cytocompatibility formulation issues [77]. Morover, S-protected gellan gum proved to be a promising mucoadhesive polymeric excipient for preparation of therapeutically potent vaginal films, providing a sustained release of metronidazole. S-protected gellan gum presented *in situ* gelling effects resulting on three-fold adhesive properties on vaginal mucus for an extended period of time (~3 h). Therefore, it is a multipurpose excipient characterized by considerable swelling properties, providing >87% cell viability, and simultaneously presenting antimicrobial effects [78].

### 5.5. Excipients Used in Devices for Vaginal Administration: Vaginal Rings

Several vaginal rings are currently available in the market or undergoing review for many treatments such as management of menopausal symptoms (atrophic vaginitis, hormonal supplementation, etc.), to be used as contraception/pregnancy maintenance or for HIV and HSV-2 prevention [79]. These products are consisted of a vaginal ring made of either silicone, ethylene vinyl acetate copolymer (EVA), thermoplastic polyurethane (TPU), polylactide, or polycaprolactone and could release the active ingredient(s) as a reservoir or matrix type systems [80]. Drug release could be sustained from a few days up to one year [79].

Eudragits^®^ are methacrylic acid copolymers; amongst them, Eudragit^®^ L100 (methacrylic acid-methyl methacrylate copolymer (1:1)) is a functional delayed release polymer for unique precise drug targeting in the gastrointestinal tract; drug release in mid to upper small intestine (dissolution at pH > 6.0); increases solubility of poorly soluble drugs in amorphous solid dispersions; sustained release matrix former for weak basic drugs with high solubility in the stomach and decreasing solubility at higher pH. In a recent study, a group of scientists developed a prototype intravaginal ring for the treatment of bacterial vaginosis. This matrix system consisted of a mixture of ethylene vinyl acetate and methacrylic acid-methyl methacrylate copolymer loaded with 150 mg DL-lactic acid with an L/D-lactic acid ratio of 1:1. The results indicated a drug release duration of seven days [81].

### 5.6. Excipients Used in Nanomedicine for Vaginal Administration

Nanoparticles (NPs) have been used as advanced DDSs due to their capability to protect therapeutic agents, their versatility to control drug release profiles and their tunable surface properties. NP encapsulation is being investigated for therapeutic applications in oral, nasal, transdermal, brain, and cardio-vascular systems as their sub-micron size allows them to penetrate into the tissue through interstitial spaces to be readily taken up by the cells. Their other advantages (modified drug release, nontoxic for the vaginal physiological environment) render them unique carriers for vaginal drug delivery [18].

Eudragits^®^ are pH-sensitive polymers used in pharmaceutics in various applications, including enteric coating materials and drug delivery vehicles [82]. When used in combination with other polymers they can alter drug release. More in particular, two different groups of researchers have studied Eudragit^®^ RS100 in the formation of clotrimazole-loaded cationic nanocapsules. One group prepared the nanocapsules, using Eudragit^®^ RS100 and medium chain triglycerides, as polymer and oily core, respectively. Results indicated that the use of this cationic polymer prolonged drug release [83]. The other group prepared a hydrogel containing clotrimazole-loaded nanocapsules, using Eudragit^®^ RS100 and two mucoadhesive polymers: Pemulen^®^ TR1 and Pullulan. Their results indicated increased mucoadhesiveness and sustained drug release [84]. In another study, Eudragit^®^ S100, composed of methacrylic acid and methyl methacrylate (1:2, MW ~ 135,000), was chosen as a pH-sensitive polymer owing to its unique dissolution behavior above pH 7.0 and employed to achieve controlled drug release. The biocompatible pH-sensitive nanoparticles, composed of Eudragit^®^ S100, showed a retained release profile at vaginal pH, rendering them a potential carrier for not only topical delivery, but also systemic delivery of therapeutically active compounds [85]. Another group of researchers produced NPs using PLGA/Eudragit^®^ S100 for the controlled delivery of tenofovir or tenofovir disoproxil fumarate intravaginally. Results indicated pH-responsive release of the microbicides [86].

Researchers have also used polyethylene oxide (PEO) polymer for the preparation of drug-loaded PEO fibers, in order to develop a formulation for bioadhesive delivery, with associated inherent advantages of high surface area for both adhesion and release. Results indicated that the release from the drug-loaded fibers had comparable rates of progesterone dissolution to that of Cyclogest^®^ 200 mg (Actavis, Barnstaple, UK), a commercially available progesterone vaginal pessary, allowing release over an extended time period (~220 min) [87].

PLGA is one of the most widely used biodegradable polymers used NPs composition. Its use is advantagious as it shows lower toxicity than non-degradable polymers, reduces immunogenic response, increases blood circulation lifetimes, protects against degradation, enhances tissue penetration, and provides sustained drug release. Recently, scientists have investigated drug delivery through biodegradable polymeric nanoparticles using this co-polymer. In particular, researchers have studied the ability of PLGA nanoparticles to encapsulate the naturally occurring chemokine regulated upon activation, normal T expressed and secreted (PSC-RANTES), and their ability to enhance tissue permeability and drug targeting. Results indicated that the encapsulated PSC-RANTES in a drug delivery PLGA nanoparticle showed increased tissue uptake, permeation, drug targeting to the site of action, and drug release over prolonged periods of time [88]. Also, researchers have developed a gel form intravaginal nanomedicine for the targeted delivery of saquinavir (SQV) for the prevention from HIV infection. The SQV-encapsulated nanoparticles were prepared from PLGA and results indicated enhanced retention within the vaginal tract [89]. In another study, scientists have combined two antiretroviral drugs, tenofovir and efavirenz, for the development of topical anti-HIV microbicides. A new vaginal delivery system was produced comprising the incorporation of drug loaded PLGA nanoparticles into a polymeric film base for prolonged release of the active ingredients [90]. Furthermore, scientists have developed PLGA nanoparticles containing raltegravir and efavirenz incorporated into a thermosensitive gel suitable for vaginal delivery. Results showed sustained drug release for both active ingredients from the nanoparticles and indicated that the developed gel-nanoparticle could have been potential for long-term vaginal pre-exposure prophylaxis for the prevention of heterosexual HIV transmission [91]. Another group of researchers developed a nanoparticle-releasing nanofiber delivery platform for optimal interaction with the vaginal mucus. More in particular, they designed mucoadhesive fibers for better retention in the vaginal tract, and PEGylated nanoparticles that diffuse quickly through mucus. Results demonstrated sustained nanoparticle release to 3d and drug retention to 7d after a single administration [92]. Therefore, PLGA nanoparticles are optimum nano-carriers, which can be applied in drug-loaded vaginal delivery systems, are mucus interactive, feasible in structural modifications and contribute to drug transfer determining its release rate.

Poloxamers are non ionic triblock copolymers composed of a hydrophobic chain of polyoxypropylene (poly(propylene oxide)) and two hydrophilic chains of polyoxyethylene (poly(ethylene oxide)). The most important characteristic of poloxamer solutions is their liquid phase, at low temperature, and the gel formation at higher temperatures. Scientists produced a nanoemulsion that was further developed in a thermosensitive gel with poloxamer 407 for vaginal itraconazole and tea tree oil application in individuals prone to recurrent vaginal candidiasis and other microbial infections. *In vitro* and *in vivo* studies showed prolonged mucoadhesion and bioadhesion properties [93]. Another research group has prepared SLNs and developed them further into a mucoadhesive and thermosensitive itraconazole gel. Stearic acid and compritol 888 (1:1, *w*/*w* ratio) were used as lipids; a mixture of 3% poloxomer 188 and 0.5% sodium taurocholate was used as surfactant, while carbopol 934 and pluronic F 127 were used for the development of the gel. Results indicated modified *in vitro* drug release of 62.2% within 20 h. *In vivo* evaluation showed prolonged bioadhesion, high tolerability, and enhanced antifungal efficacy. The formulation was successfully tested in an animal infection model and is expected to deliver similar treatment outcomes in patients suffering from candidiasis [94]. Consequently, self-assembling and gelling temperature-dependent properties of Poloxamer are potent in designing and formulating vaginal delivery systems, as they resemble in the micro-environment physical conditions of vaginal tract; thus, release rate of drug-loaded Poloxamer vehicles can be easily determined for modified release systems.

Table 1 summarizes the excipients used in modified release vaginal formulations (tablets, suppositories, gels, films, devices, and nanomedicine) in recent studies. Efficient drug delivery can be achieved with enhanced bioadhesivness and mucus penetrating substances.

## 6. Conclusions

Topical drug delivery is site specific, leading to better patients’ compliance and to the diminution of side effects. Efficient drug delivery at the vaginal cavity is often complicated, due to its discrete physiological features. Thus, researchers have sought to develop formulation strategies using novel excipients for the successful drug delivery. Biomaterials that are biocompatible and biodegradable are suitable for the formulation of novel targeted DDSs; thus, ameliorating drug-tissue compatibility issues and drug therapeutic levels. Biomaterials have gained ground in fabricating DDSs, enhancing and optimizing drug release rate, by targeting to controlled-release or time-dependent release rate. Materials that show a diversity in pH response, swelling and bioadhesive properties, similar to vaginal epithelium, along with low toxicity effects, have a significant impact on the design of multifunctional pharmaceutical formulations. To this end, several biocompatible polymers, such as chitosan, hydroxypropylmethylcellulose, methacrylic acid co-polymers, polyethylene oxide, polylactic-co-glycolide, etc., have been employed to promote mucoadhesiveness, and therefore drug release, with very promising results.

## Figures and Tables

**Table 1:** An overview of the excipients used in modified release vaginal formulations.

Formualtion/ Release Rate *	API(s)	Excipients Used	Reference
Solid dosage forms: tablets			
controlled	tenofovir	CS, pectin, locust bean gum, MS	[45]
fast and extended (double layer)	lactobacillus cells	lactose monohydrate, maize starch, ascorbic acid, stearic acid, NaCMC (1500–4500), sodium citrate dehydrate, glucose anhydrous, talc, MS, carbopol^®^ 934, CS	[46]
controlled	metronidazol	CS-NAC-MNA (S-protected CS)	[47]
controlled	clotrimazole	HPMC, NaCMC, GG, MS, dicalcium phosphate	[48]
Solid dosage forms: suppositories			
sustained	floconazole	HPMC K100, agar, PG, gelatin, glycerin, cocoa butter, bees wax	[51]
sustained	genes HeLa and HEK293	PEI (MW: 25 kDa), Suppocire^®^ BM pellets, polysorbate 80	[52]
Semi-solid dosage forms: gels			
≈6 h	acyclovir, ciprofloxacin HCl	CS citrate (medium MW and acetylation degree of 10%)	[69]
controlled	tenofovir	GG, sesame oil, sorbitan monostearate 60, polysorbate 60	[70]
sustained	metronidazole	HPMC K4M, Carbopol 934P, PCL, triethanolamine, PVA	[71]
Films			
>60 min	dapivirine	PEO N10 and N80, HPC, PEG 400, PEG 4000, vitamin E acetate	[72]
>60 min	pyrimidinedione IQP-0528	PVA-403, glycerin, PEG 400, HPMC	[73]
extended	PSS	PVA (MW: 30–70 kDa, 89–98 kDa) HEC, HPMC K4M, sorbitol, PEG 600, PG, triacetin	[74]
controlled	tioconazole	CS (MW: 230 kDa), HPMC K15M, liquid and solid vaseline, PEG 400	[75]
controlled	tenofovir	karaya gum, pectin, xanthan gum, CS (viscosity: 37 mPa⋅S, degree of N- deacetylation: 54.7 ± 4.2%), ethylcellulose	[76]
extended	tioconazole	HPMC (MW ∼ 250 kDa), CS (MW ∼ 230 KDa; 80.6% of *N*-deacetylation), PEG 400, PG	[77]
prolonged *in situ* gelling system	metronidazole	S-protected gellan gum: cysteamine and 2-MNA (2-mercaptonicotinic acid)	[78]
Devices: ring			
7 days	DL-Lactic acid	EVA 28, Eudragit^®^ L100	[81]
Nanomedicine			
prolonged cationic nanocapsules	clotrimazole	Eudragit^®^ RS100, sorbitan monooleate 80, polysorbate 80	[83]
prolonged hydrogels containing clotrimazole-loaded nanocapsules	clotrimazole	Pemulen^®^ TR1, pullulan, Eudragit^®^ RS100, sorbitan monooleate 80, polyssorbate 80, methylparaben and propylparaben, triethanolamine, imidazolidinyl urea	[84]
controlled pH-sensitive NPs	model compounds: sodium fluorescein and nile red	Eudragit^®^ S100, PVA (MW: 30–70 kDa)	[85]
controlled pH-responsive NPs	tenofovir	PLGA (MW: 76-116 kDa), Eudragit^®^ S100, poloxamer 407	[86]
prolonged mucoadhesive nanofibers	progesterone	NaCMC (MW: ~250 kDa), PEO (MW: ~200 kDa)	[87]
controlled NPs	PSC-RANTES	PLGA	[88]
sustained release NPs	saquinavir	PLGA, PVA (MW: 31–50 kDa), MES, coumarin-6, EDC, NHS	[89]
prolonged NPs in film	tenofovir and efavirenz	PLGA (MW: ~17 kDa), poloxamer 407, HPMC E4M, PVA (MW: 30–70 kDa)	[90]
sustained release NPs loaded gel	raltegravir and efavirenz	PLGA, poloxamer 407, poloxamer 188, PVA (MW: 88 kDa)	[91]
retained mucoadhesive fibers PEGylated NP fast		PVA (MW: ~105 kDa) and PVP (MW: ~1.3 MDa) PLGA, poloxamer 407, rhodamine-B, agar	[92]
prolonged muco-and bio-adhesion thermosensitive gel (nanoemulsion)	itraconazole, tea tree oil	poloxamer 407, PEG-8 caprylic/ capric glycerides, triisostearin PEG-6 esters, ethoxydiglycol	[93]
prolonged mucoadhesive and thermosensitive gel SLNs	itraconazole	poloxamer 188 (MW: 162.23 Da), stearic acid, compritol 888 (1059.8 Da), sodium taurocholate	[94]
controlled release nanoparticles, loaded nanofiber hybrid system	benzydamide	PVP, CS (MW: 50,000–190,000 Da), HPMC K100M	[95]

* Release rate as stated by the author(s). CAP: cellulose acetate phthalate, CG: carrageenan, CS: chitosan, CS-NAC-MNA: chitosan, N-acetyl-cysteine and 6-mercaptonicotinamide, EDAC: N-hydroxysuccinimide and 1-ethyl-3-(3-dimethylaminopropyl) carbodiimide hydrochloride, EDC: coumarin-6, 1-Ethyl-3-(3-dimethylaminopropyl) carbodiimide, Eudragit^®^ L100: methacrylic acid-methyl methacrylate copolymer (1:1), Eudragit^®^ RS100: poly(ethyl acrylate-co-methyl methacrylate-co-trimethylammonioethyl methacrylate chloride) (1:2:0.1), Eudragit^®^ S100: methacrylic acid and methyl methacrylate (1:2), EVA 28: ethylene: vinyl acetate (72:28%, *w*/*w*), GG: guar gum, HA-CYS-MNA: hyaluronic acid-L-cysteine ethyl ester preactivated with 6-mercaptonicotinamide, HPC: hydroxypropyl cellulose, HPMC: hydroxypropylmethyl cellulose, MCC: microcrystalline cellulose, MES: 2-(N-morpholino) ethanesulfonic acid, MS: magnesium stearate, MTT: 3-(4,5-dimethylthiazol-2-yl)-2,5-diphenyltetrazoliumbromide, NaCMC: Sodium carboxymethylcellulose, NHS: N-hydroxysuccinimide, NPs: nanoparticles, PCL: poly-ε-caprolactone, PEG: polyethylene glycol, PEI: polyethylenamine, Pemulen^®^ TR1: Acrylates/C10-30 Alkyl Acrylate Crosspolymer), PEO: polyethylene oxide, PG: propylene glycol, PLGA: polylactic-co-glycolide, PSS: polysodium-4-styrene sulfonate, PVA: polyvinyl alcohol, PVP: polyvinyl pyrrolidone, SLNs: solid lipid nanoparticles.
